# Does prophylactic antibiotic administration for tooth extraction affect PT-INR in patients taking warfarin?

**DOI:** 10.1186/s12903-020-01326-w

**Published:** 2020-11-19

**Authors:** Eiji Iwata, Akira Tachibana, Junya Kusumoto, Naoki Takata, Takumi Hasegawa, Masaya Akashi

**Affiliations:** 1 Department of Oral and Maxillofacial Surgery, Kakogawa Central City Hospital, 439 Hon-machi, Kakogawa-cho, Kakogawa, 675-8611 Japan; 2 Department of Oral and Maxillofacial Surgery, Kobe University Graduate School of Medicine, Kobe, Japan

**Keywords:** Antibiotics, Analgesics, Warfarin, Tooth extraction, International normalized ratio

## Abstract

**Background:**

Various antibiotics and analgesics have been reported to interact with warfarin. Reports that investigate the effects of medication taken for just a few days during tooth extraction on the prothrombin time-international normalized ratio are rare.

**Methods:**

A total of 110 patients receiving long-term stable warfarin therapy underwent tooth extraction without interruption of warfarin treatment. INR values were measured 1 month before the tooth extraction, the day of the extraction, and 1 week after the extraction. We investigated the changes in INR values between the day of extraction and 1 week after extraction, as well as the various risk factors for increases in INR values.

**Results:**

Before and after tooth extraction, the number of patients taking cefcapene pivoxil, amoxicillin, and azithromycin was 57, 36, and 8, respectively. Nine patients were administered ampicillin before tooth extraction and received amoxicillin after their tooth extraction. One week after tooth extraction, the INR values increased beyond the therapeutic range in 3 out of 110 patients (2.7%). The INR values before tooth extraction in these three patients were close to 3.0. The INR value increased by more than twice as much in 1 out of 110 patients (0.9%).

**Conclusion:**

Our results suggest that prophylactic antibiotic administration has little effect on INR values when patients on stable warfarin therapy undergo tooth extraction. Surgeons have to take attention if the patients whose INR values are close to 3.0 before their extraction.

## Background

Various antibiotics and analgesics have been reported to interact with warfarin [1–5]. When tooth extraction is performed, patients are usually prescribed antibiotics to prevent surgical site infection (SSI) or infective endocarditis (IE) and analgesics to decrease pain. These drugs may also interact with warfarin and produce a clinically significant alteration in anticoagulation status. However, most previous reports were on long-term treatment cases and cases where oral intake was not possible [1–5]. To our knowledge, there is only one report that investigated the effects of medication that was administered for just a few days for tooth extraction on the prothrombin time-international normalized ratio (PT-INR) [6]. This study investigated the effect of azithromycin (AZM) on INR values in patients taking warfarin [6].

In the present study, we retrospectively investigated the effects of various antibiotics administered during tooth extraction on the INR values in patients who were on stable warfarin therapy.

## Methods

### Patients

In this study, inclusion criteria were set as patients above 18 years old, and exclusion criteria were set as patients who hoped non-participate after the publication of this study. From January 2014 to December 2019, 110 patients taking warfarin underwent tooth extraction at Kakogawa Central City Hospital. Before tooth extraction, all patients consulted with their primary physicians regarding their general medical status and whether their warfarin therapies had been stable. If their INR values were over 3.0, they were advised to postpone the extraction, according to the Guidelines for Patients on Antithrombotic Therapy Requiring Dental Extraction’15 and the Guidelines for Pharmacotherapy of Atrial Fibrillation [7, 8]. In the past, several studies reported that INR values increased beyond the therapeutic range 1 week after oral administration of antibiotics [9]. Based on these reports, at Kakogawa Central City Hospital, patients taking warfarin routinely had their INR values measured on the day of their tooth extraction and remeasured those them again 1 week later. In this study, patients whose warfarin therapy was unstable or patients whose PT-INR values were above 3.0 on the day of extraction were excluded.

###  Medication

The types (e.g., cefcapene pivoxil [CFPN-PI], amoxicillin [AMPC], AZM), and dose of antibiotics were chosen at the discretion of the physicians; these were taken 1 h before tooth extraction and for a few days after extraction to prevent SSI. If the patients had valvular disease, ampicillin (ABPC) was administered 30 min before extraction to prevent IE. This was in accordance with the Guidelines for Prevention and Treatment of Infective Endocarditis (Japanese Circulation Society 2008, 2017) [10]. For analgesics, only acetaminophen (APAP) was prescribed for several days.

### Surgical procedures

All patients continued taking warfarin and were hospitalized from the day of their tooth extraction to the following day. Tooth extraction was performed under local anesthesia, administered as 1.8–3.6 mL of 2% lidocaine containing 1/80,000 units of epinephrine. The teeth were extracted by a rotation and traction movement with forceps or elevators. If immediate hemostasis was not achieved with dry gauze compression for 5 min, then a hemostat composed of oxidized cellulose (surgical; Ethicon, Somerville, NJ, USA), sutures with 3-0 absorbent thread, or a surgical splint was used at the surgeon’s discretion. After confirming hemostasis during the tooth extraction, all patients were instructed to bite down on the gauze for a few hours in the hospital room. In all patients, the presence of hemorrhage was checked by surgeons a few hours after tooth extraction. If the hemorrhage required additional treatment, such as re-suturing, we defined it as a post-extraction hemorrhage.

### Variables

We investigated the rates of the changes in the INR values beyond the therapeutic range 1 week after tooth extraction. Additionally, the following variables from medical records were retrospectively reviewed: (1) medication—types; (2) patient details in terms of—sex, age, warfarin dose, hypertension, cerebral infarction, antiplatelet therapy status (single or dual), preoperative non-steroidal anti-inflammatory drugs (NSAIDs) being taken, serum creatinine levels, estimated glomerular filtration rates (eGFR), and alanine transaminase (ALT) levels; and (3) surgical details including the—number of extracted teeth and whether they had a post-extraction hemorrhage.

## Results

### All patients

Before and after tooth extraction, the number of patients taking cefcapene pivoxil, amoxicillin, and azithromycin was 57, 36, and 8, respectively. Nine patients received ampicillin before—tooth extraction and received amoxicillin after—tooth extraction.

### Patients who took CFPN-PI and APAP

From January 2014 to June 2016, CFPN-PI was the primary antibiotic used to prevent SSI. All 57 patients received 300 mg of CFPN-PI per day for 3 days before and after tooth extraction, followed by 1200–1800 mg of APAP per day for 3 days after tooth extraction. Seven out of the 57 patients underwent additional treatments due to post-extraction hemorrhages on the day of extraction.

**Fig. 1:**
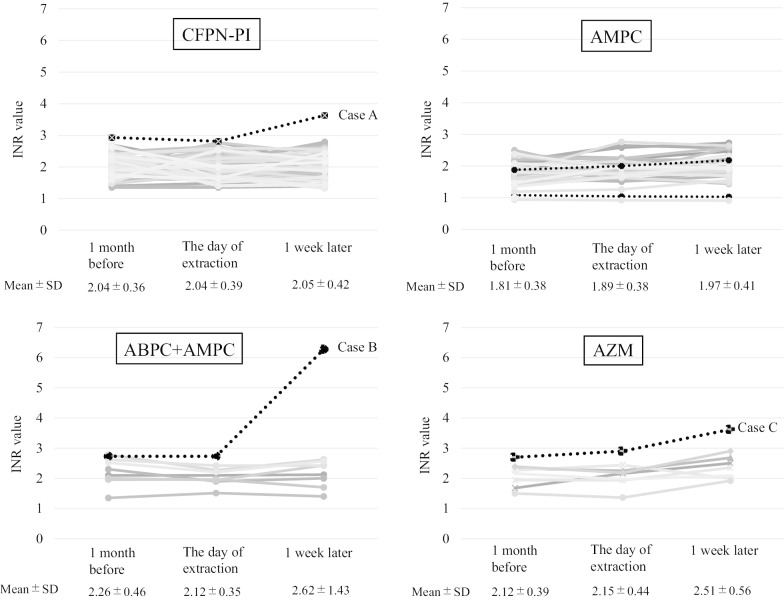
Change of INR value with time. The changes in INR throughout the study were not statistically significant. (2-factor analysis of variance, NS)

The INR values increased beyond the therapeutic range in 1 out of 57 patients (Fig. 1). We present this patient as Case A. Case A was a 76-year-old female taking warfarin 1.75 mg per day. A preoperative blood test showed an ALT value of 11. The patient took 300 mg of CFPN-PI per day for 3 days, followed by 1800 mg per day for 3 days, similar to other patients, and had 12 teeth (the greatest number of teeth of the 57 patients) extracted. The patient’s INR value was within the therapeutic range, but was the highest out of all 57 patients, with a value of nearly 3.0 1 month before tooth extraction and on the day of the extraction (2.93 and 2.81, respectively) (Fig. 1).

###  Patients who took AMPC and APAP

From July 2016 to December 2019, AMPC was the main antibiotic prescribed, as opposed to CFPN-PI. Most of the 36 patients took AMPC at a dose of 750 mg/day for 2 days (two patients took if for 3 days) before and after tooth extraction, and APAP at a dose of 1200 mg/day for 3–7 days after tooth extraction. One patient underwent additional treatments due to post-extraction hemorrhages on the day of extraction.

###  Patients who took ABPC, AMPC and APAP

Most of the nine patients were treated with a single 2-g dose of ABPC the day before tooth extraction, and then took AMPC at a dose of 750 mg/day for 2 days (just one patient took it for 4 days), and 1200–1500 mg/day of APAP for 3–7 days after tooth extraction. Two out of the nine patients underwent additional treatments due to post-extraction hemorrhages on the day of extraction, and one out of two patients had a post-extraction hemorrhage after discharge.

One week after tooth extraction, the INR value in one patient increased by more than twice as much (Fig. 1). We present this patient as Case B. Case B was a 66-year-old female taking warfarin 4.0 mg per day. A preoperative blood test showed an ALT value of 10. The patient had two teeth extracted and was treated with a single 2-g dose of ABPC the day before tooth extraction, and then took AMPC at a dose of 750 mg/day for 2 days like the other patients. The patient had a post-extraction hemorrhage on the day of the extraction and visited our hospital due to re-post-extraction hemorrhage 2 days after tooth extraction as well. After the patient’s hemostasis was treated, she was prescribed AMPC for 2 days and APAP for 3 days. In addition, the patient’s INR value was within the therapeutic range, but had a value close to 3.0 both 1 month before tooth extraction and on the day of the extraction (2.73 and 2.73, respectively) (Fig. 1).

### Patients who took AZM and APAP

AZM was mainly used for patients with impaired renal function or penicillin allergy. All eight patients took AZM at a dose of 500 mg/day for 3 days before and after tooth extraction, and 1200–1500 mg/day for 3–7 days after tooth extraction. None of the patients had post-extraction hemorrhages.

One week after tooth extraction, a patient’s INR value increased beyond the therapeutic range (Fig. 1). We present this patient as Case C. Case C was a 78-year-old male taking warfarin 2.25 mg per day. A preoperative blood test showed an ALT value of 12. The patient had one tooth extracted and took AZM at a dose of 500 mg/day for 3 days, followed by 1200 mg/day of APAP for 7 days. However, while Case C’s INR was within the therapeutic range, it was the highest of all eight patients in this group, with a value close to 3.0 both 1 month before tooth extraction and on the day of the extraction (2.70 and 2.90, respectively) (Fig. 1).

## Discussion

In the present study, we investigated the effects that various antibiotics and analgesics taken during tooth extraction had on the INR values of patients taking warfarin. Initially, we classified the patients into two groups (INR value increasing group and decreasing group after tooth extraction) and compared these two groups. However, there were no significantly different factors (e.g., warfarin dose, number of extracted teeth and type or dose of antibiotics) between the two groups (Additional file 1: Table S1). In addition, the increase/decrease values were mostly within 20% of the INR before extraction, which is within the range of error. Therefore, we focused on the fact that the medication before and after tooth extraction hardly raised the INR value sharply and we presented three patients in which the value increased beyond the therapeutic range. The INR values before tooth extraction in these three patients were close to 3.0. The INR value increased by more than twice as much in 1 of 110 patients (0.9%).

According to the Guidelines for Patients on Antithrombotic Therapy Requiring Dental Extraction 15, tooth extraction can be safely performed without interrupting warfarin when the INR value is below 3.0 [7]. In the present study, when the INR values were below 3.0 in all patients, there were no cases of hemorrhages that required systemic treatment (e.g., vitamin K or clotting factor), and no thromboses (e.g., cerebral embolisms). Post-extraction hemorrhage that required additional treatment such as re-suturing, was observed in seven out of 110 patients (6.4%) (Table 1). In the past, many reports have investigated post-extraction hemorrhages in patients taking anticoagulants, and reported that the incidence of post-extraction hemorrhages was 0–26% [11–15]. Our results were similar to those of other reports [11–15].

**Table 1:** Patient data

Variables		
Types of antibiotics	CFPN-PI	57 (51.8)
	AMPC	36 (32.7)
	ABPC + AMPC	9 (8.2)
	AZM	8 (7.3)
Sex	Male	73 (66.4)
	Female	37 (33.6)
Age	Mean ± SD	72.5 ± 9.1
	< 75	59 (53.6)
	≥ 75	51 (46.4)
Warfarin dose (mg)	Mean ± SD	2.72 ± 1.10
Diabetes mellitus	No	85 (77.3)
	Yes	25 (22.7)
Hypertension	No	57 (51.8)
	Yes	53 (48.2)
Cerebral infarction	No	93 (84.5)
	Yes	17 (15.5)
With antiplatelet therapy	No	82 (74.5)
	Single	27 (24.5)
	Dual	1 (1.0)
Preoperative NSAIDs	No	106 (96.4)
	Yes	4 (0.6)
Serum creatinine (mg/dl)	Mean ± SD	0.93 ± 0.30
eGFR (mL/min/1.73 m^2^)	Mean ± SD	60.0 ± 16.9
ALT (IU/L)	Mean ± SD	21.2 ± 13.8
Number of extracted teeth	Mean ± SD	2.3 ± 2.2
	Single tooth	54 (49.1)
	Multiple teeth	56 (50.9)
Post-extraction hemorrhage (having additional treatment)	No	103 (93.6)
	Yes	7 (6.4)

Values are expressed as absolute numbers, with the corresponding percentage of the total in parentheses. Some variables are expressed as the mean ± standard deviation in a parametric ratio scale

Some reports have shown that an increased age (> 75 years old), male, high doses of warfarin, renal failure or liver failure, diarrhea, and drug interactions can all cause increases in INR values [16, 17]. Regarding the interaction between antibiotics and warfarin, Rice et al. [1] conducted a review of many reports and reported that many antibiotics increased INR values, although the duration of administration varied. The mechanism by which antibiotics increase the action of warfarin is known to alter the intestinal flora and decrease the production of vitamin K, thereby enhancing the action of warfarin. This mechanism was applicable to cephalosporins (e.g., CFPN-PI) and penicillin antibiotics (e.g., AMPC). Antibiotics also inhibit cytochrome P-450 (CYP) in the liver, increasing the concentration of warfarin in the blood [1]. This mechanism was applicable to macrolide antibiotics (e.g., AZM). No reports have investigated the effect of surgical invasion by tooth extraction on the INR. Several studies have shown that infection and inflammation decrease the expression and activity of CYP, resulting in decreased drug clearance [1, 3]. Other studies have reported that infection itself affects the metabolism of warfarin [3, 4]. These reports may suggest that tooth extraction affects the INR values. In the present study, Case B, whose INR value more than doubled 1 week after tooth extraction, was the only one patient who underwent additional treatments for two post-extraction hemorrhages. Case B might have been the most invasive case and had evidence of an infection accompanied by necrotic tissue and delayed wound healing 1 week after tooth extraction.

Several reports have investigated the relationship between various antibiotics and INR values [6, 9, 18]. Ghaswalla et al. [18] reported that there was a significant interaction between time and antibiotics on the INR values of elderly (> 65 years old) patients who were on stable warfarin therapy (CFPN-PI, AMPC, AZM, levofloxacin [LVFX]). AZM in particular, which has a significantly long half-life, has been widely discussed in this context [6, 9]. Glasheen et al. reported that the INR value increased beyond the therapeutic range in 31% of AZM cases and 33% of levofloxacin LVFX cases 1 week after oral administration. This was seen in patients on stable warfarin therapy [9]. On the other hand, Kusafuka et al. [6] reported that changes in INR values 1 week before and after tooth extraction were not statistically significant (2-factor analysis of variance, not significant [NS]) when 18 patients taking warfarin were administered AZM. In the present study, the changes in INR values throughout the study were not statistically significant (2-factor analysis of variance, NS) for all antibiotics, including AZM. However, in 3 patients whose INR values were close to 3.0 before tooth extraction, the INR values increased beyond the therapeutic range. This result indicates that surgeons have to pay attention to medications when the INR value is close to 3.0 before the tooth extraction.

Although most NSAIDs are known to enhance the action of warfarin [5], APAP also requires discussion [19]. Cardeira et al. [19] conducted a review of many reports and reported that taking APAP was associated with a mean INR increase of 0.62 compared to placebo, for patients taking warfarin. However, in all reports used in this review, the duration of APAP treatment was longer than 4 weeks. In the present study the APAP treatment was just for 3–7 days after tooth extraction, so there may have been no significant association between APAP and the increase in the INR values 1 week after extraction. Surgeons need to be wary about prescribing APAP long term.

This study has some limitations. First, there is a possibility of unknown confounding factors and factors not studied (e.g., the presence of diarrhea) due to the retrospective nature of this study. Second, the number of patients was small depending on the antibiotics prescribed. Third, frequent PT-INR measurements are highly invasive for patients, so we have to set criteria for measurements based on patients. In the present study, the INR value increased beyond the therapeutic range in 3 out of 110 patients (2.7%). The INR values were close to 3.0 before tooth extraction in these three patients. Our results suggest that surgeons have to take precautions before performing tooth extraction when INR values are close to 3.0. In addition, in those cases, measuring INR values 1 week later may be useful. Finally, in our hospital, the patients have been routinely prophylactically administered antibiotics for a few days after tooth extraction. However, in recent years, we have been trying to reduce the administration of unnecessary antibiotic prophylaxis to prevent antimicrobial resistance.

## Conclusion

Our results suggest that prophylactic antibiotic administration has little effect on INR values when patients on stable warfarin therapy undergo tooth extraction. Surgeons have to take attention if the patients whose INR values are close to 3.0 before their extraction.

## Supplementary information

**Additional file 1: Table S1.** Comparison of the patients whose INR value increased and those whose INR value decreased after one week after extraction. Values are expressed as absolute numbers, with the corresponding percentage of the total in parentheses. Some variables are expressed as the mean ± standard deviation in a parametric ratio scale.

## Data Availability

The datasets generated and analyzed during the current study are not publicly available because it contains personal information but are available from the corresponding author on reasonable request.

## Abbreviations

PT-INR: Prothrombin time-international normalized ratio
SSI: Surgical site infection
IE: Infective endocarditis
AZM: Azithromycin
CFPN-PI: Cefcapene pivoxil
AMPC: Amoxicillin
ABPC: Ampicillin
APAP: Acetaminophen
NJ: State of New Jersey
USA: United States of America
NSAIDs: Non-steroidal anti-inflammatory drugs
eGFR: Estimated glomerular filtration rates
ALT: Alanine transaminase
CYP: Cytochrome P-450
LVFX: Levofloxacin
NS: Not significant
