# Supplementary material for: Does prophylactic antibiotic administration for tooth extraction affect PT-INR in patients taking warfarin?
Source: BMC Oral Health. 2020 Nov 19;20:331. doi: 10.1186/s12903-020-01326-w (PMC7678308; doi:10.1186/s12903-020-01326-w)
Supplement: Supplementary file 1 — Additional file 1: Table S1. Comparison of the patients whose INR value increased and those whose INR value decreased after one week after extraction. Values are expressed as absolute numbers, with the corresponding percentage of the total in parentheses. Some variables are expressed as the mean ± standard deviation in a parametric ratio scale. [file 12903_2020_1326_MOESM1_ESM.doc]

**Table S1.**

| Variables |  | | INR increase after tooth extraction | | P value |
| --- | --- | --- | --- | --- | --- |
|  |  | | + (n=63) | − (n=47) |  |
| Gender | Male | | 41 (65.1) | 32 (68.1) | 0.840 |
|  | Female | | 22 (34.9) | 15 (31.9) |  |
| Age | Mean ± SD | | 72.4 ± 9.5 | 72.6± 8.7 | 0.892 |
|  | <75 | | 34 (54.0) | 25 (53.2) | 1.000 |
|  | ≥75 | | 29 (46.0) | 22 (46.8) |  |
| Warfarin dose (mg) | Mean ± SD | | 2.77± 1.09 | 2.65 ± 1.12 | 0.558 |
| Diabetes mellitus | No | | 47 (74.6) | 38 (80.9) | 0.657 |
|  | Yes | | 16 (25.4) | 9 (19.1) |  |
| Hypertension | No | | 31 (49.2) | 26 (56.5) | 0.567 |
|  | Yes | | 32 (50.8) | 21 (43.5) |  |
| Cerebral infarction | No | | 53 (84.1) | 40 (85.1) | 1.000 |
|  | Yes | | 10 (15.9) | 7 (14.9) |  |
| With antiplatelet therapy | No | | 48 (76.2) | 34 (72.3) | 0.490 |
|  | Single | | 15 (23.8) | 12 (25.5) |  |
|  | Dual | | 0 (0.0) | 1 (2.2) |  |
| Preoperative NSAIDs | No | | 60 (96.8) | 46 (97.9) | 0.634 |
|  | Yes | | 3 (3.2) | 1 (2.1) |  |
| Serum creatinine (mg/dl) | Mean ± SD | | 0.95 ± 0.32 | 0.92 ± 0.24 | 0.657 |
| eGFR (mL/min/1.73 m^2^) | Mean ± SD | | 60.7 ± 20.1 | 59.2 ± 11.8 | 0.701 |
| ALT (IU/L) | Mean ± SD | | 21.0 ± 11.3 | 21.4 ± 16.8 | 0.891 |
| Number of extracted teeth | Mean ± SD | | 2.25 ± 1.88 | 2.36 ± 2.54 | 0.799 |
|  | Single tooth | | 27 (42.9) | 27 (57.4) | 0.248 |
|  | Multiple teeth | | 36 (57.1) | 20 (42.6) |  |
| Post-extraction hemorrhage | No | | 57 (90.5) | 46 (97.9) | 0.235 |
| (having additional treatment) | Yes | | 6 (9.5) | 1 (2.1) |  |
|  | |  | | | |
